# Supplementary material for: Structural basis of template strand deoxyuridine promoter recognition by a viral RNA polymerase
Source: Nat Commun. 2022 Jun 20;13:3526. doi: 10.1038/s41467-022-31214-6 (PMC9209446; doi:10.1038/s41467-022-31214-6)
Supplement: Supplementary file 1 — Supplementary Information [file 41467_2022_31214_MOESM1_ESM.pdf]

Supplementary Information

Structural basis of template strand deoxyuridine promoter recognition by a viral RNA polymerase

Alec Fraser, Maria L. Sokolova, Arina V. Drobysheva, Julia V. Gordeeva, Sergei Borukhov,

John Jumper, Konstantin V. Severinov, Petr G. Leiman

Contents

Full List of AlphaFold Team Members

Supplementary Figures 1-8

Supplementary Tables 1-6

Full List of AlphaFold Team Members

John Jumper,
Richard Evans,
Alexander Pritzel,
Tim Green,
Michael Figurnov,
Kathryn Tunyasuvunakool,
Olaf Ronneberger,
Russ Bates,
Augustin Žídek,
Alex Bridgland,
Clemens Meyer,
Simon A A Kohl,
Anna Potapenko,
Andrew J Ballard,
Andrew Cowie,
Bernardino Romera-Paredes,
Stanislav Nikolov,
Rishub Jain,
Jonas Adler,
Trevor Back,
Stig Petersen,
David Reiman,
Martin Steinegger,
Michalina Pacholska,
David Silver,
Oriol Vinyals,
Andrew W Senior,
Koray Kavukcuoglu,
Pushmeet Kohli,
Demis Hassabis

Figure 1c

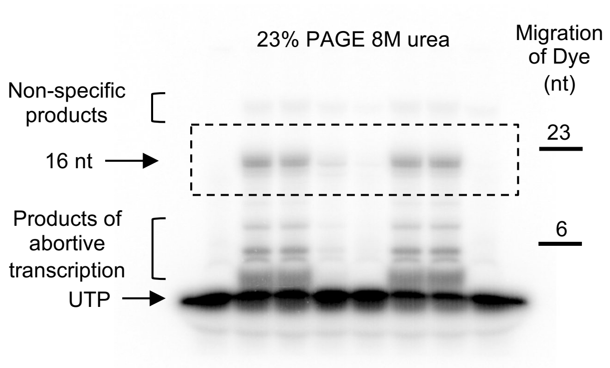

Figure 1d

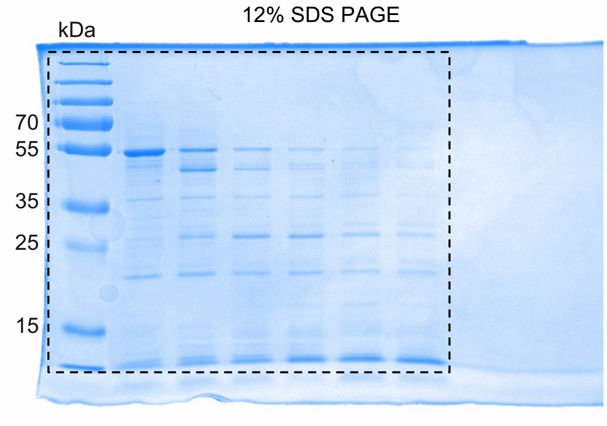

Figure 8b

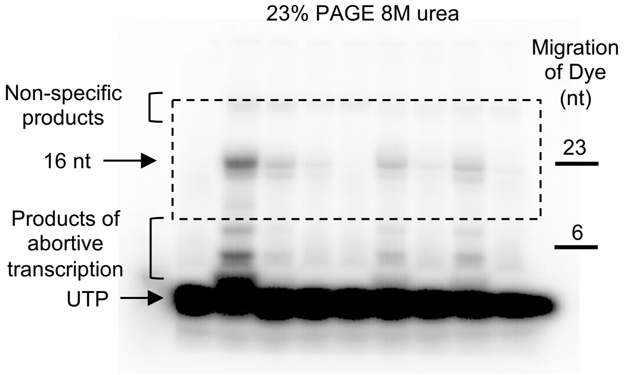

Figure 8d (upper panel)

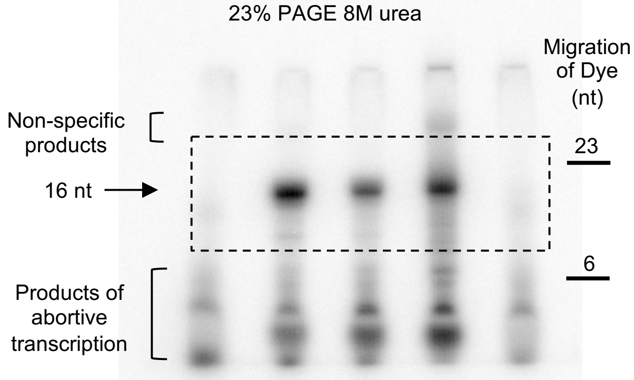

Figure 8g

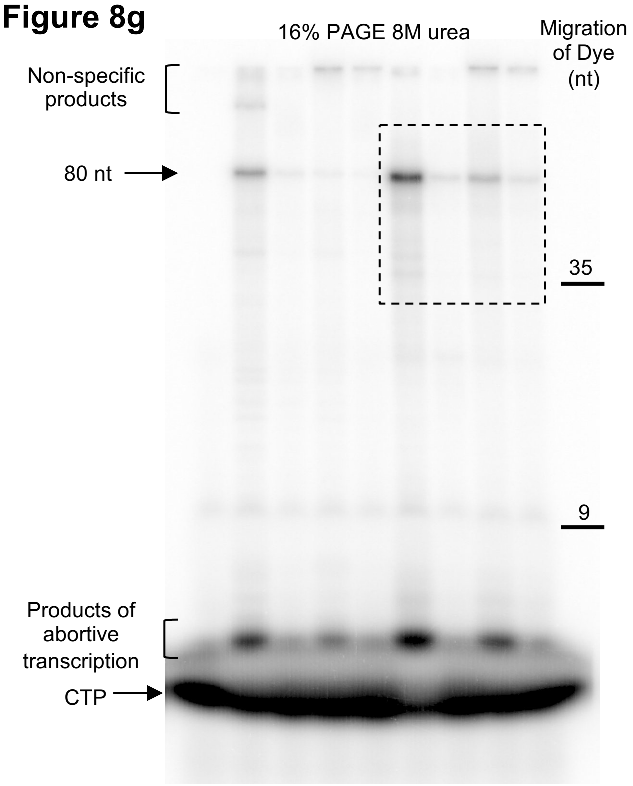

Figure 8d (lower panel)

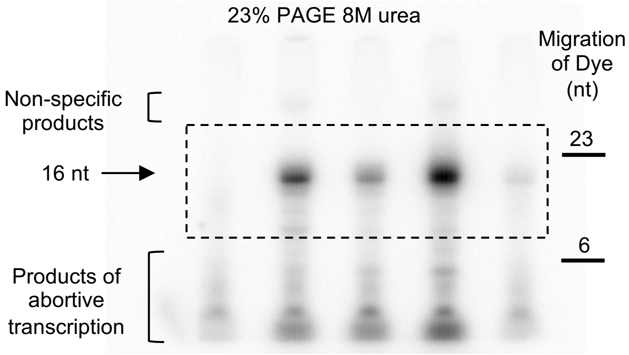

Supplementary Figure 1. Minimally cropped but otherwise unedited images of the autoradiographs and SDS PAGE used in the main text figures with the areas shown in the main text figures marked by dashed lines. The original images without any labels are presented in the **Source Data File 1**.

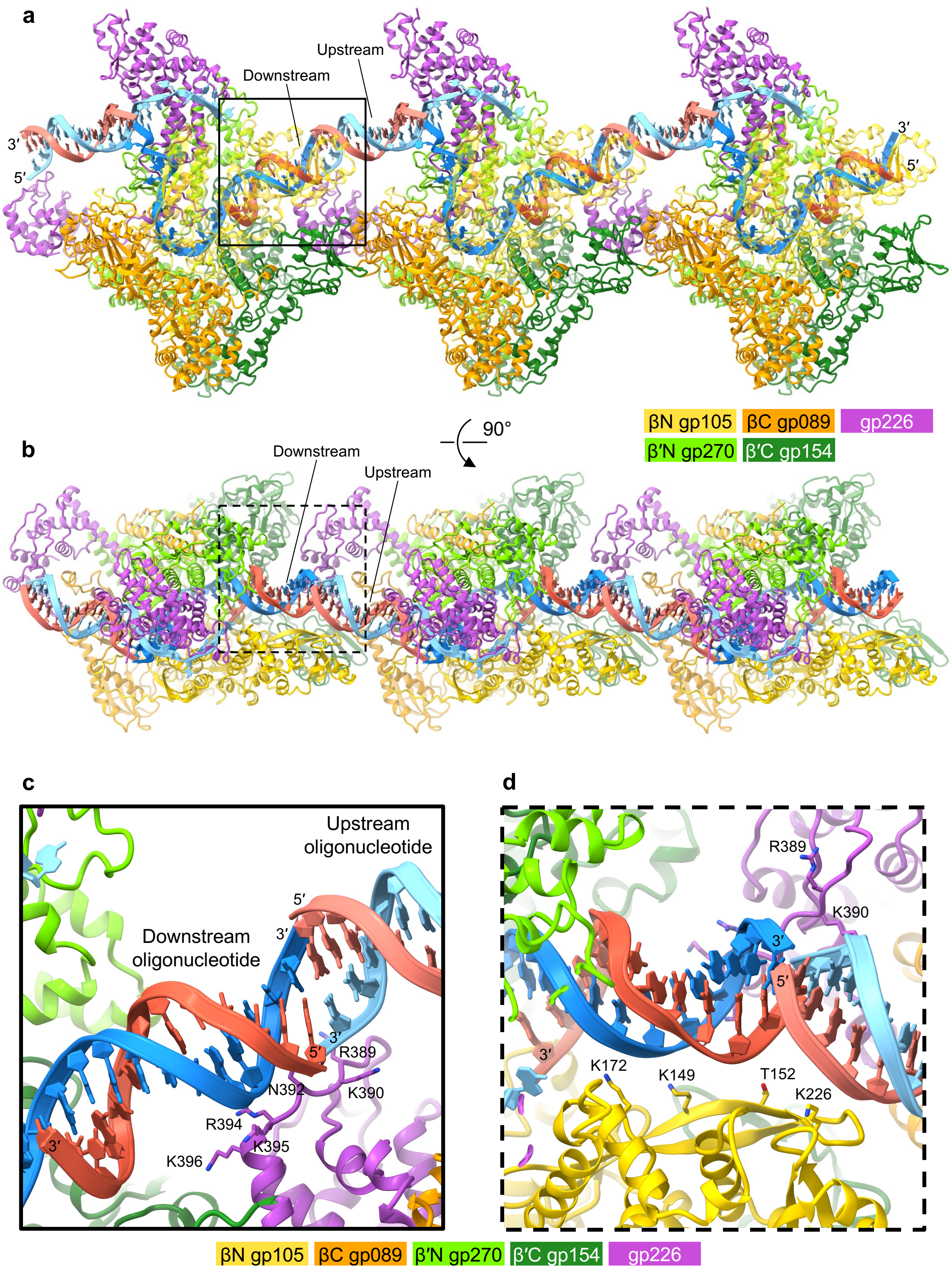

Supplementary Figure 2. AR9 nvRNAPs and up- and downstream oligonucleotides form a train *in crystallo* in the AR9 nvRNAP-Pro-Xray dataset.

a and **b**, Two orthogonal views of three unit cells demonstrating the peculiar crystal packing of AR9 nvRNAP promoter complexes (AR9 nvRNAP-Pro-Xray).

c and **d**, Two areas of interest indicated with a solid and dashed line square in panels **a** and **b** that show details of pi-pi stacking interactions between the ends of the up- and downstream oligonucleotides. Residues most proximal to the DNA are shown in a stick representation and labeled. In both panels, the color code is as in **Fig. 2a**.

Supplementary Figure 3

β-like subunits

```
E._coli_β         ....MVYSYTEKKR.I..R...KD....FGKRPQ.VLDVPY..LLSIQLD 33
AR9_gp105         .MIS.........N.F..RKF..HGNKN.....QE..KFNENLI.LNKEN 27
phiKZ_gp123       M.P...........DPFLI.EK.I.REN.....TP..CMNPTLANGITVE 28
                                                        .          :

E._coli_β         SFQ.........KFIEQDPEGQYGLE.AAF..R..S.V.........FPI 59
AR9_gp105         E...........SIL.......NYL..D..PIC..KTL..EIIPEITY.. 49
phiKZ_gp123       HTMTRDPNTGVNMTR.R.....Y.IDSL....FDIS..SVL.....F... 57
                                          :          .

E._coli_β         QSYSGNSEL....QYV..SYRLGE.......................... 77
AR9_gp105         ..............L.GSSVEPINK................VYKFNKEE 67
phiKZ_gp123       .........PDGFKYEG.NRAC..TPLKHFEEITREYNAKRI........ 87
                                    .

E._coli_β         .....PVFDVQECQIRGVTYSAPLRVKLR........LVIYEREAPEGT. 113
AR9_gp105         KTS.D..........IERSELQLIKMSFLIEKDDKKEE............ 94
phiKZ_gp123       ..ANI...........APTDMYMIDLMFS.........Y.........KG 106
                                    :    : : :

E._coli_β         VKDIKE.QEV.YMGEIPLMTDNG.TFVINGTERVIVSQLHRSPGVFFD.. 158
AR9_gp105         ......I..NK.FIYFPKLIDSQ.YFIINGNRYYPIYQLLDS.GTY.RT. 131
phiKZ_gp123       ...E.MLY.PR.PMLLPAFK.RGNMVTINGAKYIGSPVLTDVGF.S.VLN 147
                                 :* :      . *** .      *

E._coli_β         SDKGKTHSSGKVLYNARIIPYR.GSWLDFEFDPKD............NLF 195
AR9_gp105         .N...........KA.LTLKTLLMPIVLREK..KETFDDINGETHTMLNV 166
phiKZ_gp123       .D............S.IFIPFRRTKLTFK..................... 162
                   :                :

E._coli_β         VRID.RRRKLPATIILRAL....NYTT..EQ.......ILD.LFFEKVIF 230
AR9_gp105         DLDLF.KSKV.P..FLIYFFSKFGFE.GT.LEYFGLQD..LI........ 200
phiKZ_gp123       .QTDH............................................. 166

E._coli_β         EIRDNKLQMELVPERLRGETASFDIEANGKVYVEKGRRITARHIRQLEKD 280
AR9_gp105         ..................................................
phiKZ_gp123       ..................................................

E._coli_β         DVKLIEVPVEYIAGKVVAK.DYIDESTGEL.I.CAANMELSLDLLAKLSQ 327
AR9_gp105         ..................HVL........MKEDL..........D..Q.. 210
phiKZ_gp123       ..................................................

E._coli_β         SGHK..RI...ETL.............F..TNDLDH.GP..Y..ISET.L 351
AR9_gp105         ..LDED.EIND.NVIFMITKNISLVVDKNFF....SNK.NNQIIIA.TLL 250
phiKZ_gp123       ..................................................

E._coli_β         RVD.PT.......ND..R...LSALVEIYRMMRPGEP............. 375
AR9_gp105         .NCFN.TRIKIDK.IYEKDYWVKKLGGY.F...TT.NN............ 280
phiKZ_gp123       ......................................HYMCNGQRKIMY 178

E._coli_β         ..................................................
AR9_gp105         ..................................................
phiKZ_gp123       VIWSQIHNEMAKRTKRDLGNRPHIESCLAHYFFCQFGVTQTFKQWANVDV 228
```

Supplementary Figure 3

```
E._coli_β        ..................................................
AR9_gp105        ..................................................
phiKZ_gp123      KCGLLSDFPEEEYPREKWNIYSSATLKGKHPTGEMVLVIPRHQESIFATR 278


E._coli_β        .............................................PTREA 380
AR9_gp105        ............................................S.NKQEK 286
phiKZ_gp123      LIAGFWYVVDAFPMRFTRPEYVDSTNLWRVILGHMVFGDFEHQGKVE.EN 327
                                                                 *

E._coli_β        AESLFENLFF.SEDRYD....LS.AVGRMKF.NRSLLREEIEG...SGIL 420
AR9_gp105        GEGIILSF.E.R..ILD.E.W.TKKI.LR...T......E..EKNKE.DI 316
phiKZ_gp123      IDSHLHS..FCNS..L.DEMT.IEEL.KTV.GV......N....V.S.TI 357
                  :. : .                  :             :      .  :

E._coli_β        SKDDIIDVMKK.LIDIRNGKG.....EV.DDIDHL.GNRR.IRSVGEMAE 461
AR9_gp105        YS.VVRWMINNY...L....ALV....K.QDNMNL.ANKR.IRL.YEYLL 350
phiKZ_gp123      WE.LLYEIMTSLA..H......HLYA.TDIDETSMYG..KRLTV.LHYLM 394
                  .  :  ::..                   *   : .  : :    .

E._coli_β        NQFRV..GLV...RVE.R................AVK.ER........L. 479
AR9_gp105        HP.LLIK..F..S...KG.TYRV...LNNRNSNK..FEK........... 375
phiKZ_gp123      SE.FNY...AVSM..F..GY..MFQSR.............RDREWTVQEL 421


E._coli_β        SLGDLDTLMPQDM...I.......NAKPI..SAAVKEFFG.SS.QLSQFM 515
AR9_gp105        .............IK...TIFS.NIQ...EGF.LVKKI.INNEL..LRYD 401
phiKZ_gp123      .........N..E.GLKR..S.F.KL.....QTAIKRL.T.VDHGELDTM 448
                                                  :*.:    .

E._coli_β        DQNNPLSEIT.HKRRISA..LGPGG.LTRER..AG............FEV 547
AR9_gp105        NSVNSI.SLFTLILRYTQ..SGPQSPFS.SNSTNN.............KL 434
phiKZ_gp123      SNPNSS.MLIKGTSILVTQDRAK...........TAKAHNKSLINDSS. 484
                 .. *.   :            .                          .

E._coli_β        RDVHPTHYGRVCPIETPEGPN.IGLI..NSLSVYA.Q.....TNEYGFLE 588
AR9_gp105        RGLHPSYLGRLGLTSTS.AGD.PGAS..GSLTPFLELPENSYM....HF. 475
phiKZ_gp123      RIIHASIAEVGQYKNQP.K.NNPD..GRGRLNMYTKVGP..TGL....V. 523
                 * :*.:        . .   :  .    . *. :              .

E._coli_β        TPYRKVTDGVVTDEIHYLS...AIEEGNYV.....IAQANSNLDEEGHFV 630
AR9_gp105        ..................TEEP.E.......................... 480
phiKZ_gp123      ..................E..........RREEVR............... 530


E._coli_β        EDLVTC..RSKGES............SLFSRDQVDYMDVSTQQVVSVGAS 666
AR9_gp105        ......................INLN........................ 484
phiKZ_gp123      .....EIID....NAQLMFRAK............................ 543
```

Split in nvRNAP β-like subunits

```
E._coli_β          VTDGVVTDEIHYLSAIEEGNYVIA.Q.A.......N.SNLDEEGHFVEDL 633
AR9_gp89           ......................KNEDY.......EG............. 14
phiKZ_gp71-73      ...........................MSQLGRREI............. 9
                                                      :

E._coli_β          VTCRSKGESSLFSRDQVDYMDVSTQ.QVVSVGASLIPFLEHDDANRALMG 682
AR9_gp89           ......................S.H.RFLA.EELLMPNANKTDGNRSTMF 39
phiKZ_gp71-73      ......................D.LTL.LG.HTGLDPWYGTTSSARGAMF 34
                                         .     :.    * *     .. *. *
```

Supplementary Figure 3

```
E._coli_β          ANMQRQAVPTLRADKPLVGTGMERAVAVDSG.VTAV.AK...RGGVVQYV 727
AR9_gp89           CSHLAQAVTLQKAEPPLVYTNFENQVGKYSTAGYRKA.N.SN........ 79
phiKZ_gp71-73      VTHIGQAPEVNGNESRYFLTGAELEYAKY.TH.DVRF.PED......... 72
                    .   **      :   . *. *   .

E._coli_β          DASRIVIKVNEDEMYPGEAGID............................ 749
AR9_gp89           ....................Y.KVI.EKIYKN..........DYNYV.L 95
phiKZ_gp71-73      .....................CR.VLHVLRKYPTGIGKDSIRSNPVTTI 99


E._coli_β          ....................IYNLTKYTRSNQ..NTCINQM..P..... 769
AR9_gp89           IVQ.DQET.....GEYT.LFE....RAECEFLTEHYGFQW.DNDK...ID 130
phiKZ_gp71-73      I.YEN.YFDKYKT..IGVLHV....PE.YMSHHQDFGYEL.VK.NREVW. 137

                                                     β-strand 1 (DPBB)
E._coli_β          ...............CVSLGEPVERGDV.LA..DGPSTDL.GELALGQNM 800
AR9_gp89           SL.KK..DD.TI.EK..........D...TVLYKNTCYDENMNFGYGVNL 162
phiKZ_gp71-73      .ETIAPNE.M.FS.K.............DTVIAQSGAVKKDGTLGMGVNA 170
                                                 . .. .  .   :. * *

                 β-strand 1 (DPBB)  β-strand 2 (DPBB)
E._coli_β          RVAFMPWNGYNFEDSILVSERVVQEDRFTTIHIQELACVSRDTKLGPEE. 849
AR9_gp89           NAAYFSYKNETLEDAIVISESAA..KKLGTFSVNKVKVSV.......NT. 202
phiKZ_gp71-73      NVVFLSA.AGTIEDGFVANKNFL..KRMMPTSYSTAVANA.......GRK 210
                   ...::.    .:**.:: .:     .:: .   .

E._coli_β          ..ITA.DIPNVGEAAL..S.....KLDESGIVYIGAEV.TGGD.I.LVGK 886
AR9_gp89           ND.ILL.........NLYGDNENY.K...GFPDIGEHIKNQ.IIAS...R 234
phiKZ_gp71-73      AF.FLN..........MYGDDKIY.K...PFPDIGDVIRPDGVIFA...I 242
                                     .          :   ** :

E._coli_β          VTPK..GETQLTPEEKLLRAIFGEKAS...............DVKDSSL 918
AR9_gp89           RRFDYN..................TALYELK.N.LNEMR.DS...DT.P 258
phiKZ_gp71-73      RDHDDD..................LAPAEMTPRALRTLDRTF...DR.A 269
                      .                      :                  *

E._coli_β          RVPNGVS.GTVIDVQVFTRDG.VE..................KDKR.AL 946
AR9_gp89           FFA..D..GKIVDIEIFSN..VPEEELKV........QKYNEQ.VLYYIN 293
phiKZ_gp71-73      VIG..TPGAKVIDIDIWRD...E......RVNPSPTPTGMDAQ.LVKYHT 307
                    .      ..::*::::

E._coli_β          EIE.EMQL.......KQAKKDLSEELQILEAGLFSRIRAVLVAGGVEAEK 988
AR9_gp89           .KQKE..FS.NNVYQ................................... 304
phiKZ_gp71-73      .HLSS...YYR....................................... 314
                       .

E._coli_β          LDKLPRDRWLELGLTDEEKQNQLEQLAEQYDELKHEFEKKLEAKR.R... 1034
AR9_gp89           ....................................K.L........KK 308
phiKZ_gp71-73      ..........................................ELLKI. 319


E._coli_β          ................................K.ITQG.D......... 1040
AR9_gp89           ...IVE..GKD.....NNVSD.KLL..HFYN.NCK..MR..IDENI.... 336
phiKZ_gp71-73      YRG..LLARR.KDDLH...ITEEF.ERL..IVTA...Q.MF.L..PQPDN 353

                                            β-strand 3 (DPBB) β-strand 4 (DPBB)
E._coli_β          ....D.LA.....PGV.LKIVKVYLAVKRRIQPGDKMAGRHGNKGVISKI 1079
AR9_gp89           ...SY.TYQ.N..SKFSGFIMEFTILEEEPLNKGSKITGRYGNKGVISKI 379
phiKZ_gp71-73      VRKL.SRFYR.LDP.LDEWRVEVTYKAQKMPAGAFKMTDFHGGKGVICKV 400
                                . .    ::.    :.    . *::. :*.****.*:
```

Supplementary Figure 3

```
                                       β-strand 5 (DPBB)
E._coli_β          NPIEDMPYDE....NGTPVDIVLNPLGVPSRMNIGQILETHLGM.AAKG. 1123
AR9_gp89           LPDDQMPTVAEGRFKGLKADICLNPLGVFNRLNPSQLIEQELNWIA..KF 427
phiKZ_gp71-73      MEDEDMPIDE....NGNRADLIIFGGSTMRRSNYGRIYEHGFGAAA..RD 444
                      ::**       :*  .*: :   ..  * * .:: *  :.  *

E._coli_β          .IGDK...INA.........MLK...QQ..QE......VAKLRE..FIQ 1146
AR9_gp89           I...RKDM..E.E.......................AG..SN.EEKV.. 442
phiKZ_gp71-73      L...AQRL..RVEAGLDRHAK..PTQQQLNSVMGNTQ.W..V..DYAF.. 480
                                                               :  .

E._coli_β          R..A...YDL..GADVR...QKV...D.LSTFSD.EEV..M....RLA.. 1173
AR9_gp89           SILLDFL..NRVN..KEET..ELME.EFIN.SLNKT.E..L..EE..FLN 477
phiKZ_gp71-73      KELLGFY..EIIAP.T.MHS.K..MME.HP..N.....PAEHVK...TVL 512
                                        :    :

E._coli_β          ENLR..KGMPIATPVFDGA.K.E..A...EIK.......ELLKLGDLP.. 1205
AR9_gp89           D..IIENGIPICQKPFFGN.I.GLDELWE..LYNHYDH.........I.D 511
phiKZ_gp71-73      M..D.G.FPYIYAPV.DD.PVDLMAAVNK..LIN..SDK...Y....RPH 545
                             *      .
                                                  β-strand 6 (DPBB)
E._coli_β          TSGQIRLYDGR.....TG.EQFERPVTVGYMYMLKLNHLVDDK.MH.ARS 1247
AR9_gp89           ....YFKCE..........G.ISTPLIIGEIYMVRLKHEP.HSK.FSARS 544
phiKZ_gp71-73      ....YGKVSYRDQAGKW.VT.TKDNVLMGPLYMMLLEKIG.E.D.WSAAA 586
                           .             .  : :* :**: *::     .     * :

E._coli_β          TGSYSL.VT......QQPLG............GKAQFGGQRF.GEMEVWA 1277
AR9_gp89           TSFM.NLR......GL.PAKS.KNFKEHKDLY.S..KTPVR.IGNMEISN 581
phiKZ_gp71-73      SVKT.Q...PFGLP.S.KLNNADRA...STPG.R..ETAIRSFGESETRS 624
                   :                                       *  *: *

E._coli_β          L.EAYG.AA..YTLQ.EMLTVKSDDVNGRTKMYKNIV.DG....N.HQM. 1315
AR9_gp89           L.SL.TN.EMGS.IMDM.LNSYSNNETNRRELIMQLLTGN.PFDT.NI.D 623
phiKZ_gp71-73      YNCT.V..GPGP.TAEI.LDQT.NNPLAHAAVIESWLTAEKPSSVPVAVD 668
                                     *    ::   :  :  .  :

E._coli_β          E......PG.MPESF.NVLL..KEIRSLGINIE....LE......DE 1342
AR9_gp89           L..S.D..VES.GTS.KILK..SLFTC.LGLS.....IDDV...... 649
phiKZ_gp71-73      REKIPFG.G.S.RPVAM..FDHL........LECSGIALEYAPDH.. 700
                                .
```

β′-like subunits

```
E._coli_β′      .....MKDLLKFLKAQTKTEEFDAIKI....................... 22
AR9_gp270       MGKKL....................S........................ 6
phiKZ_gp55      ...........................MGSSHHHHHHSSGLVPRGSHMGL 3


E._coli_β′      ...ALASPDM.IRSWS........FGEVKKP.........ETIN.YRTFK 50
AR9_gp270       ...L.IDFNEI..Y.N......EENL.ITRAN........P.IENH..E. 30
phiKZ_gp55      YAKV.VDHNEV..H..DQFTGKRI.YAND...YNTSNSDEK..E...... 36
                      . :                                  :

E._coli_β′      PE...RDGLFC.ARIF.GPV..K.DYECLCGKYKRLKHRGVICEKCGVEV 92
AR9_gp270       FS...DDGIYSER..IFG.SYNEDD......................... 49
phiKZ_gp55      .EFDRH.FYS........................................ 44
                 .

E._coli_β′      TQTKV.RRERMGHIEL..A.SPT........................... 111
AR9_gp270       ..DDKD.IDTIGWINIEPYY.II........................... 68
phiKZ_gp55      .......................HFQDSEAIESSVSCDCRAIEDAHKLGV 71
```

Supplementary Figure 3

```
E._coli_β'      ..................................................
AR9_gp270       ..................................................
phiKZ_gp55      ICDICNTPVVNTSSRPIEPSMWVRTPKHVRSLINPRLIIMLTGYLVTKEF 121

E._coli_β'      .............................AHIWFLKSLPSRI..GLLLD 129
AR9_gp270       ............................N.PI.LFT...I.IKKC.... 79
phiKZ_gp55      DFLAYLTDTSYRYDVESIGSKETRRKVDRLLHR.G.......FER..... 158
                                                          :

E._coli_β'      MPLRDIE....R.VLYF.............ESYVVIEGGMTNLERQQILT 161
AR9_gp270       I...PSI....NKIINYQQSIDQNGENIDL.................... 102
phiKZ_gp55      ......GLNHFI.DN................................... 166

E._coli_β'      EE....QY...LDALEEFGDEFDAKMGAE..AIQ.A..LL.KSM...... 192
AR9_gp270       .TEEIG.EDDY..............IG.LVKF...KDNF....DDLLEKY 128
phiKZ_gp55      .........................FN.EI....FQ....FL.LD..AN. 178
                                         :.

E._coli_β'      ...DLEQECEQLREELNETNSETKRKK....LTKRIKL.LEAF..VQSGN 232
AR9_gp270       TDK......................K.K.Y..QK..EYD..FLIE.... 143
phiKZ_gp55      II.........................SNNK..SE..FAQ..FVAQ...N 194
                                                 ..      *
                                              β' lid
E._coli_β'      KPEWMIL..T.VLPVLPPDLRPLV...PLDGGR......FATSD..LNDL 268
AR9_gp270       NHDKIFI..N.KLPVFSHKLRPATL..L....TGSKGKVLAF.DE.INNY 182
phiKZ_gp55      K.DK..LFPKYL.PVPSKLCFVA..EST....TS....GTYL.DKPI..E 227
                : :   :  .   ** .                          *  :

E._coli_β'      YRRVINRN.NRLK..RLLD.....L...........AAP..DI.IV... 292
AR9_gp270       YNFVIE.YINQ.INE...GVVSDD............S.I.DL.L.L..L 208
phiKZ_gp55      AAIDAT...L...............TFASIDASSVPLS..PI....KAQN 253
                                                     :
                                         β' rudder
E._coli_β'      RNEKRM..L..QEAVDALLDNGRRGRAITGSNKRPLKSLAD.MIKGKQGR 337
AR9_gp270       P.LLYN..M..QFYANNILT.................RIISEYLRGKKGF 236
phiKZ_gp55      ...R..TMRGLR.LYGQFYE.................IYAKSRIAQKPGL 280
                           :   . :                      .  :  * *
                          β-strand 1 (DPBB)  β-strand 2 (DPBB)
E._coli_β'      FRQNLLGKRVDYSGRSVITVGPY.LRLHQCGLPKKMALELFKPFIYGKLE 386
AR9_gp270       LRKNIMGSRINFSARNVITPLIG.HPIDEVAMPYKTFAELYKFQLINLIS 285
phiKZ_gp55      ARRHMFGARLNATARAVITSISDPHDYDELHIPWGVGCQLLKYHLTNKLK 330
                 *::::* *:: :.* ***        .:  :*     :* *  : . :.
                                                       β-strand 3 (DPBB)
E._coli_β'      LRGL.ATTIKAAKKMVERE....EA.VVWDILDEVIR......EHPVLLN 424
AR9_gp270       KV.KG.INYNEALKFWEKGILGF.NQELYNYMEELITKTK.G.GCTFLLN 330
phiKZ_gp55      AK.FN.MTTREAFSFVYENVLQY.NQIIADLFKELIAEAAPYKGMGCTFH 377
                       . . * .:  .         : : :.*:*            ::
                                                       Catalytic β-strand 6
                        β-strand 4 (DPBB) β-strand 5 (DPBB) motif   (DPBB)
E._coli_β'      RAPTL.HRLGIQAFEP.VLI.E..GKAIQLHPLVCAAYNADFDGDQMAVH 469
AR9_gp270       RNPTISI.GSILYLKIGLIKKDYKDLTLGISNNLLSALSGDYDGDVLNII 379
phiKZ_gp55      RNPTLQR.GSTQQFFITKVKDDINDNSISMSVLCLKAPNADFDGDQLNLT 426
                * **:    .   :    :  :  . :: :      * ..*:*** : :

E._coli_β'      VPLTLEAQLEARAL.MMSTNN.I.LSPAN..G..EP.IIVPSQDVVLGLY 511
AR9_gp270       PVFDNKMKEHF..SLLSP.QNFL.VDR.N.NG.RFNGDFDLQKDQILGIF 422
phiKZ_gp55      LMPDVYLTKAT..ERIAP..HT.WVLSIDEP.HEISGNLELQGPVVETII 470
                               : .  :   :   :         :  .   :  :
```

Supplementary Figure 3

```
E._coli_β'      YMTRDCVNAKGEGMVLTGPKEAERLYRSGLASLHARV..........KVR 551
AR9_gp270       ILNN.............................................. 426
phiKZ_gp55      NWAH.............................EKYLPPLEEWLKAK... 488
                   .
```

Split in nvRNAP β′-like subunits

```
E._coli_β'      IVPSQDVVLGLYYMTRDCVNAKGEGMV..LTG.................. 529
AR9_gp154       ...........................MEKTYNL.NDI.LLSNEYE.KIK 20
phiKZ_gp74      .........................MN..LNRYKARDLLN....LSYDDL 19
                                              .

E._coli_β'      ..PKEAERLY......R.................SGLAS..L.HARVKVR 551
AR9_gp154       ED.I......KEEIINDMASKKVKYSNTSEFAKN...DFLKDE...F.ID 56
phiKZ_gp74      WS.LPS...................................E...W.HL 28

E._coli_β'      ITEYEKDANGELVAKT...SLKDTTVGRAILWM..I.VP..........K 585
AR9_gp154       LVV.........DGE...TYE.I...TYGNLITLLIVAR.PFNHFKVPM 88
phiKZ_gp74      IEF.D..........DGKTVV.S...VDRITKLSVLCWYPLK.HY.K.. 58
                :                  :               :

E._coli_β'      ...G.LP..YSIVN................QAL.G.KKAI.SKMLNTCYR 610
AR9_gp154       ..TEDLLF...DL.................SDL.....K...EYQNYYTT 108
phiKZ_gp74      DCPIP.SDHH..IDFNRILTDNPKDYLNVEGGRVTSK..AMVKHLNKAIW 103
                            :                             :  *

E._coli_β'      ILG..............LK....PTV..IFADQIM..YTGF..AYAA.R 634
AR9_gp154       .LLE.HF....G.YS.NE.IK..SI.IKDV.ISELAI.F.SG..DINV.T 140
phiKZ_gp74      NI.YDW.SGETVDP.EV...LSKL..AIEG.KNWLYNQT..TVKL..SEY 140
                 :                              . :

E._coli_β'      SGASV...GIDDMV........IP.EK..KHEIISEAEAEV.AEIQEQFQ 669
AR9_gp154       FGNTV...SIKSLIDLGNKVKR.FRELLHYR..LP..NDEAL........ 174
phiKZ_gp74      .LATLSMFDIAEVYN.H...PK.VREA..NHNI..EP............. 167
                   ::    .* .:            *    :

E._coli_β'      SGLVTAGERYNKVIDIWAA.ANDRVSKAMMDNLQT.ETVINRDGQEEKQV 717
AR9_gp154       ............EFNDIEA.IIKKNLDEIMKILSE............... 196
phiKZ_gp74      ...............TT.YGIEKISYGKVKEVFNDP.............T 188
                                      .     :  . :.

E._coli_β'      SFN..SIYMMADSGARGSAAQIRQLAGMRGLMAKPDGSIIET.PITANFR 764
AR9_gp154       TDN.ML.RYYIDSGAGINSKQFGQVLSLVGSKPDLFGKIIPY.PINTSFL 243
phiKZ_gp74      QFIGNSIIEGLRSGT.QKTEQLLQAFAWRGFPTDIN.SDIFKYPVTTGYI 236
                            **:  .: *: *  .  *  ..   .  *   *:.:.:
```

Bridge Helix

```
E._coli_β'      EGLNVLL..QYF.ISTHGARKGLADTA...LKTA.NSGYLTRRLVDVAQDL 807
AR9_gp154       RGL.DVR.S.FYINALGARKALITNY...QQVR.NSGYLTRKISMLLMDT 286
phiKZ_gp74      DGIWNLYEN.M.IESRSGTKALL..YNKEL..LRVTEYFNRKSQLIAQYV 280
                 *:  :  . : *.: .. *.*             : *:.*:
```

Zn-binding cysteine 1

```
E._coli_β'      V.VTED....D.CGT..H.EGIMM.TPVIEGGDVKE.PLRDRVLGRVTAE 846
AR9_gp154       K.L.ID...LDDCGSH.ENNYLSINVEN.....K.DV.L.KRFSKR.SYL 321
phiKZ_gp74      QRL..HPGD.CK.T..TI.LAEY.PVTK.......LT.L.KAFKGK.YY. 311
                  :  .                    .            * . .  :

E._coli_β'      DVL.KPGTADILVPRN....T.LLHEQWC...DLLEE.NSVD....AVKV 882
AR9_gp154       .NN.............NGELVE.I....DI.N...D...ESLIGQV.IKI 344
phiKZ_gp74      QKED.......G.K......LD.W....IRGN...E.T.H.LIGTK.QKF 335
                                                   :            *.
```

Supplementary Figure 3

```
                 Zn-binding cysteines 2, 3, and 4
E._coli_β'      RSVVSCDT....DFGVCA.HCYG.R.DLARGHII...N...KGEAIGVIA 919
AR9_gp154       PSPTTCAS....NEGVCR.KCYGKL.......FDI.NK.D...LNIGMIA 377
phiKZ_gp74      RSV.FGC.NHPDSQG.ICMTCYG.RLG.......INI.PKG..TNIGQVA 371
                 *          . *     ***                      ** :*
                 Trigger Loop helix 1
E._coli_β'      AQSIGEPGTQL..TMRT..FHIGGAASR.AAA.ESSIQVKNKGSIKLSNV 963
AR9_gp154       VLLLTDPLT.QRLL..SAKH..L.LETRS.S.K................. 402
phiKZ_gp74      AVSMGDKIT.SAVL.................................... 384
                .  : :  *

E._coli_β'      KSVVNSSGKLVITSRNTELKLIDEFGRTKESYKVPYGAVLAKGDGEQVAG 1013
AR9_gp154       ..................................................
phiKZ_gp74      ..................................................

E._coli_β'      GETVANWDPH..............................TMPVITEVSG 1033
AR9_gp154       .........IDWGTNFEENFIVNRNLIYPKVYNGTVIIKE.......... 433
phiKZ_gp74      ..................................................

                       Trigger Loop Insertion Domain
E._coli_β'      FVRFTDMIDGQTIT..............RQTDELT...GLSSLVVLDSAE 1066
AR9_gp154       .........D...DFKEDEETEEQVFDT.FTL.KSGNRFISI........ 461
phiKZ_gp74      ..................................................

E._coli_β'      RTAGGKDLRPALKIVDAQGNDVLIPGTDMPAQYFLPGKAIVQ.LEDGVQI 1115
AR9_gp154       .........................................SS....... 463
phiKZ_gp74      ..................................................

E._coli_β'      SSGDTLARIPQ....................................... 1126
AR9_gp154       .........P.MRLFLNKDLKKQLDESFYNIEEMQFEIPLNKLDEGDSFA 503
phiKZ_gp74      ..................................................

E._coli_β'      .....ESGGT........................................ 1131
AR9_gp154       TFIMD..NNEL....................................... 512
phiKZ_gp74      ...........STKHTDASSAVEQYKLGKIESNYLRTGEIPETLYLKKEL 423

E._coli_β'      ..................................................
AR9_gp154       ..................................................
phiKZ_gp74      TQKDYRLVIARSEAENLADILMIDDLTAYPATSATELTSLALVYDDEVNG 473

E._coli_β'      ..................................................
AR9_gp154       ..................................................
phiKZ_gp74      ECGDVLTVSLYNRRASLSIEMLKHIKMVRWELDQRDNIVISLRGFDFNLP 523

                                                Trigger Loop helix 2
E._coli_β'      ............................KDITG..GL.PRVA.DLF.EA. 1147
AR9_gp154       ............................S....KPLR..EIK.DLIETN. 526
phiKZ_gp74      FLTLPNKHVNMYEVMKRFQSFLHSGSDSA...E...AGKL.STE.KVGYT 565
                                                   :

E._coli_β'      ...RRPKEPAILAEISGIVSFGKETKGKRRLVITPVDGSDPYEEMIPKWR 1194
AR9_gp154       ....K............................................. 527
phiKZ_gp74      SKTY.............................................. 569
```

```
E._coli_β′      QLNVFEGERVERGDVISDGPEAPHDI.L.RLRGVHAVTRYIVNEVQ.DVY 1241
AR9_gp154       .....................YIKDH.N.....VNEVVNYFIYL.LNES. 548
phiKZ_gp74      ......................LK..NYK...SPIEALPVFATMAN.EKI 591
                                       :            .   :      :

E._coli_β′      RLQGVKINDKHIEVIVRQMLRKATIVNAGSSDFLEGEQVEY......... 1282
AR9_gp154       G...INIQSVHSELIIREMMKLD.................D.....S.. 570
phiKZ_gp74      ....S.LNISHCEILIYA.MMI.................RSAQYRDYRL 617
                      ::  * *:::   :

E._coli_β′      S..RVK........IANRELEANGKVGATYS.RDLLGITKASLAT...ES 1318
AR9_gp154       DRT..Q.FKNDKMP...............D.YEIFRITDANLKG...DS 597
phiKZ_gp74      PKP...GI.....N................GQFEK....Y.NRLMQCRS 637
                                                                 *

E._coli_β′      FISAASFQETTRVLT.E...AAVAG.K.RD.......ELRGLKENVIV.. 1353
AR9_gp154       LSRSLLFEQVKKQLTTLDYDTF....NK.T......K..SSILDK.LL.. 631
phiKZ_gp74      LGGAMAFE.K..QH..E...PLNNPGS...FLNKMRN..DHPYDL.LVKG 673
                :  :  *:            .     .                :  ::

E._coli_β′      GR...LIPAGTGYAYHQDRMRRRAAGEAPAAPQVTAEDASASLAELLNAG 1400
AR9_gp154       ..................................................
phiKZ_gp74      .GKLR............................................. 677

E._coli_β′      LGGSDNE 1407
AR9_gp154       .......
phiKZ_gp74      .......
```

Supplementary Figure 3. Structure-based sequence alignment of *E. coli* RNAP, AR9 nvRNAP and phiKZ nvRNAP calculated using UCSF Chimera.

Conserved, catalytic site-proximal structural elements are shown in the **Fig. 2** color scheme. Residues comprising the β-strands of the double-ψ β-barrel (DPBB) domains are highlighted in bold. Zn-binding motifs are colored gold. Residues absent from the atomic models are in grey color.

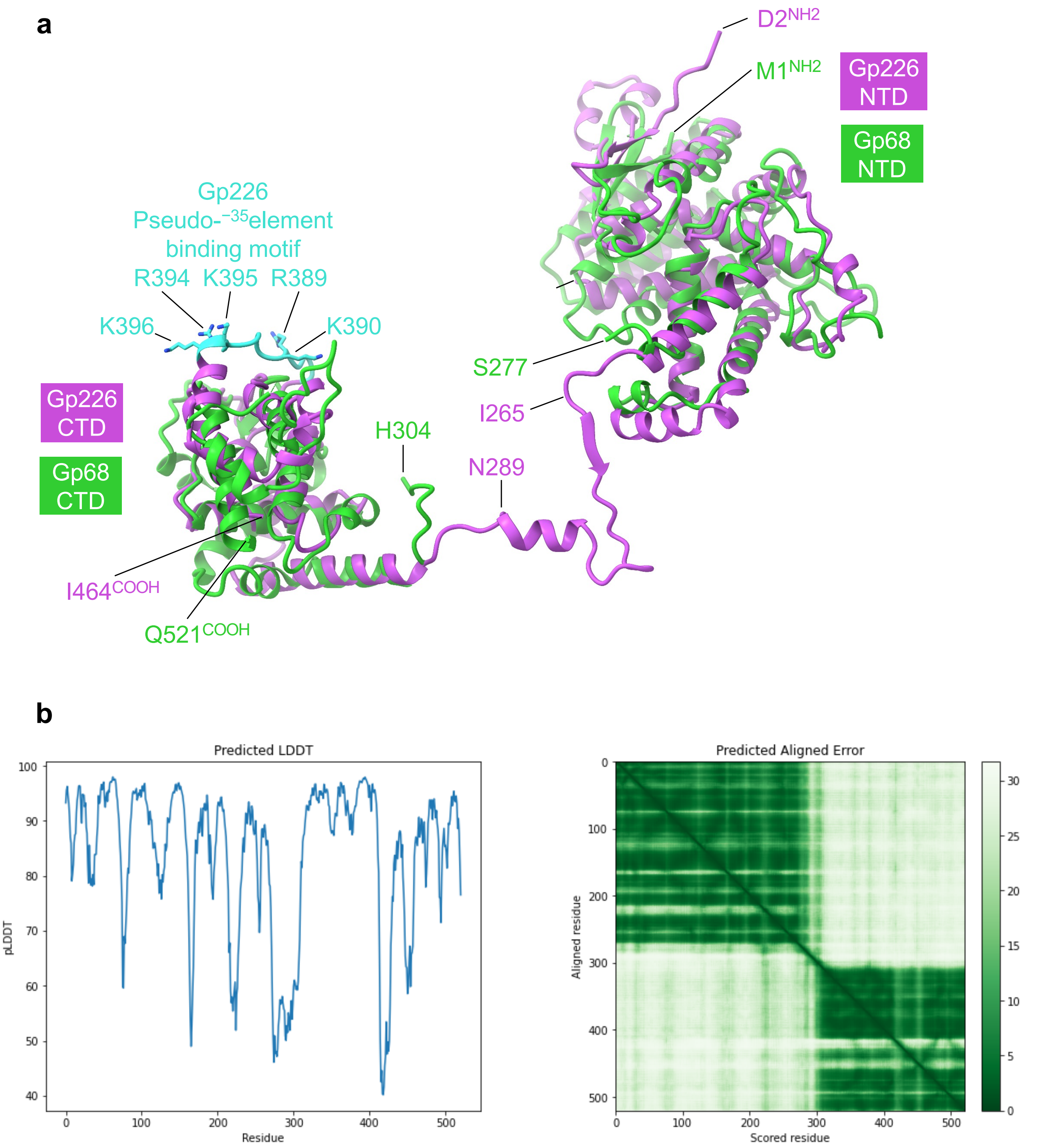

Supplementary Figure 4. Comparison of AR9 gp226 and phiKZ gp68 structures.
a, Superposition of phiKZ gp68 NTD and CTD onto gp226. The structure of gp68 NTD is predicted by AlphaFold Colab using default parameters (the atomic coordinates are given in the **Source Data File 2**). The structure of gp68 CTD is taken from the cryo-EM structure of phiKZ nvRNAP holoenzyme (PDB code 7OGP). The orientation of AR9 gp226 is as in **Fig. 6**. Residue identities and numbers are given at strategic locations.
b, Quality factors of the gp68 model created by AlphaFold Colab. The entire sequence of gp68 was used. The accuracy of the gp68 NTD model (residues 1-277) can be evaluated by comparing the AlphaFold model of the gp68 CTD (residues 304-521) with its cryo-EM structure. The latter two can be superimposed with an RMSD of 1.2 Å for 191 equivalent Cα atoms (out of 203 ordered in the cryoEM structure).

Supplementary Figure 5

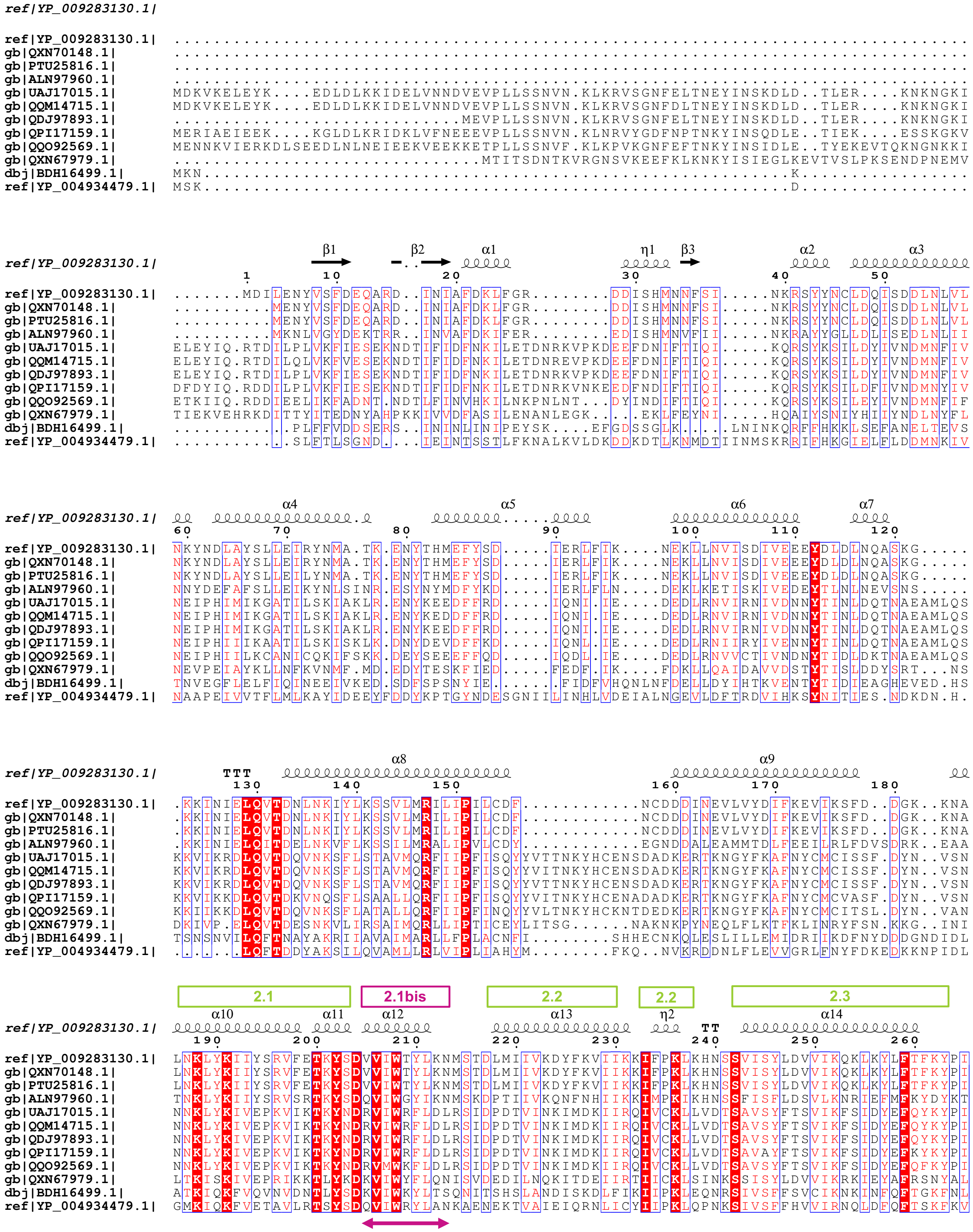

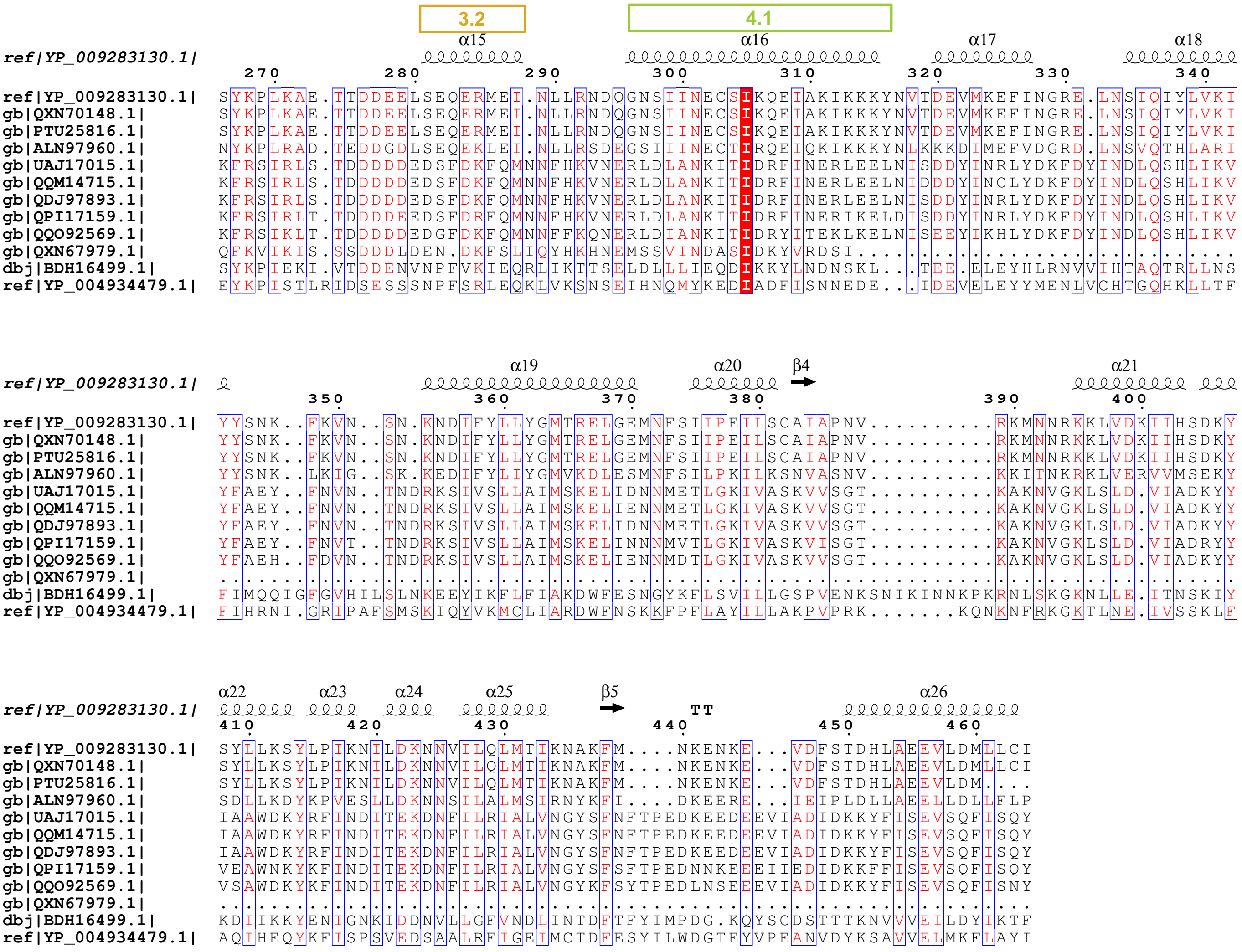

Sequences used in the alignment

Accession	Description
YP_009283130.1	DNA-directed RNA polymerase subunit [Bacillus phage AR9] (**gp226**)
QXN70148.1	putative DNA directed RNA polymerase subunit [Bacillus phage vB_BspM_Internexus]
PTU25816.1	hypothetical protein DA469_21760 [Bacillus subtilis]
ALN97960.1	hypothetical protein Bp8pS_281 [Bacillus phage vB_BpuM-BpSp]
UAJ17015.1	hypothetical protein UFVDC4_00088 [Staphylococcus phage vB_SauM-UFV_DC4]
QQM14715.1	non-viral RNA polymerase subunit [Staphylococcus phage MarsHill]
QDJ97893.1	hypothetical protein PALS2_268 [Staphylococcus phage PALS_2]
QPI17159.1	hypothetical protein [Staphylococcus phage vB_StaM_SA1]
QQO92569.1	non-viral RNA polymerase specificity subunit [Staphylococcus phage Machias]
QXN67979.1	hypothetical protein FPHOBKDP_00229 [Listeria phage LPJP1]
BDH16499.1	hypothetical protein [Bacteriophage sp.]
YP_004934479.1	hypothetical protein phiR1-37_gp245 [Yersinia phage phiR1-37]

Supplementary Figure 5. Sequence alignment of gp226 orthologs currently available in GenBank (as of March 30, 2022) identified using a BLAST search.

The σ-like elements are labeled and colored using the nomenclature and color scheme of **Fig. 6**. The fragment shown in the **inset** of **Fig. 6a** is highlighted with a plum-colored double arrow.

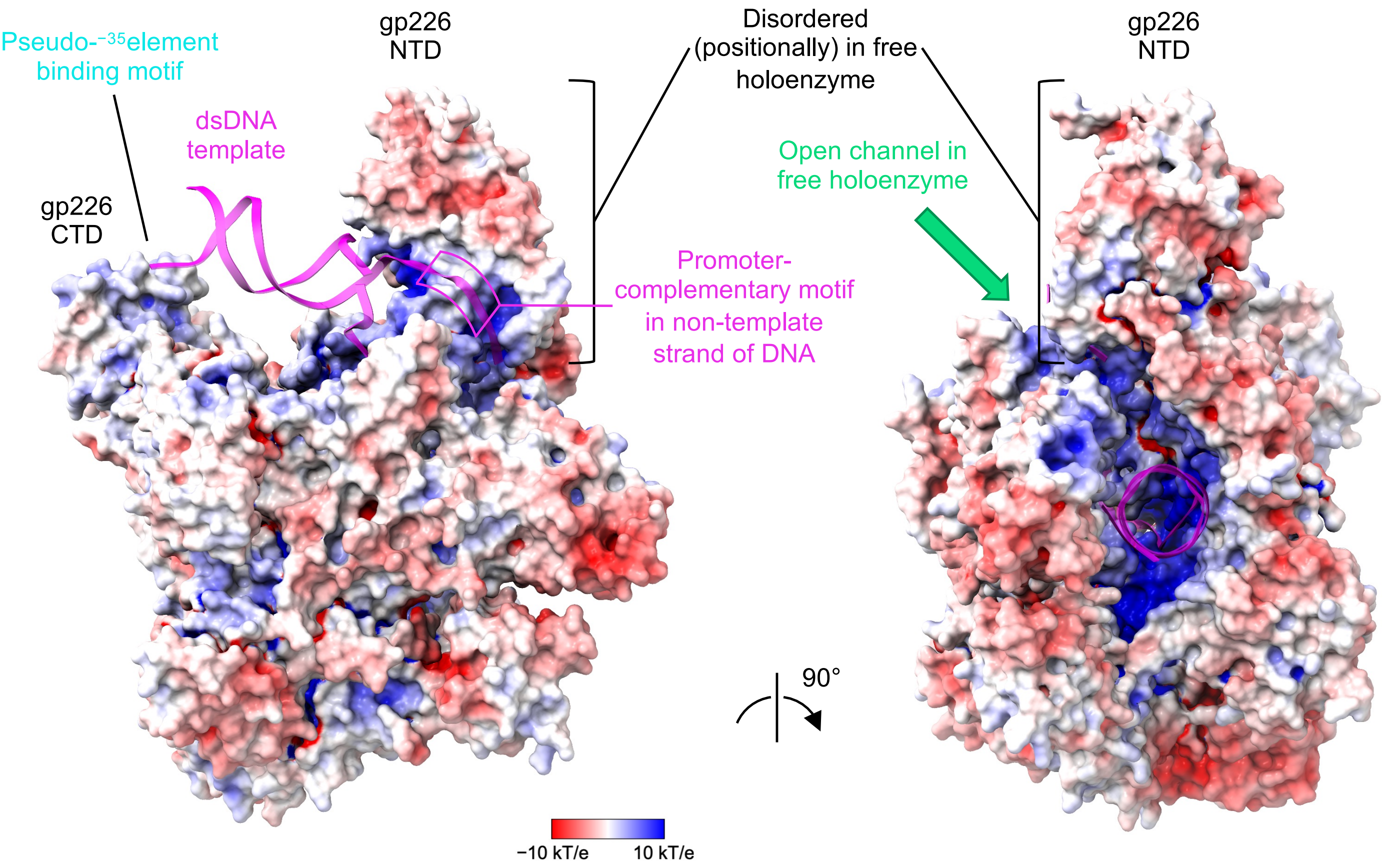

Supplementary Figure 6. Distribution of electrostatic potential on the molecular surface of the AR9 nvRNAP promoter complex.
The DNA (colored magenta) was excluded from the calculations. The orientation of the molecule in the left panel is as in **Fig. 2a**.

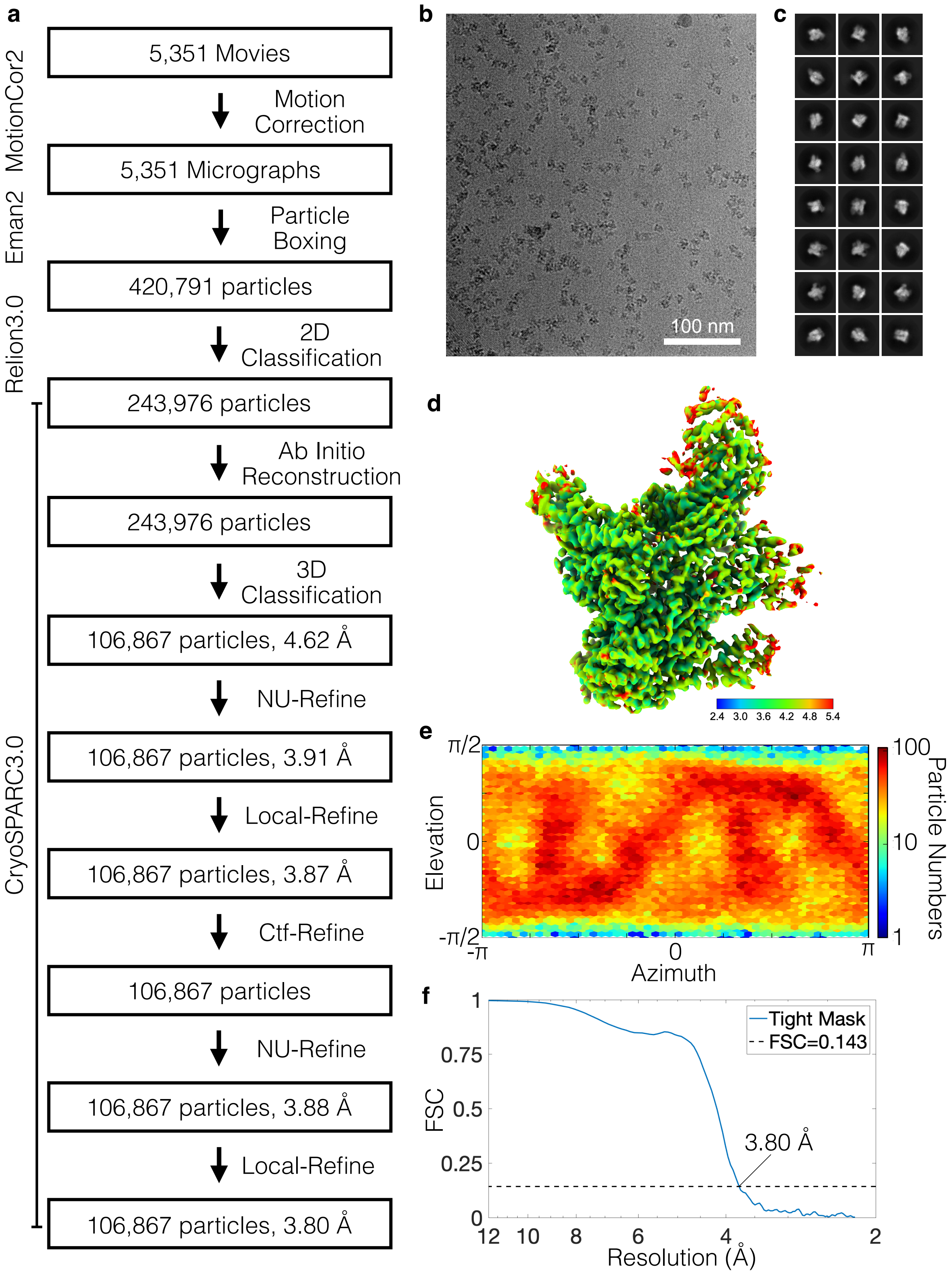

Supplementary Figure 7. Cryo-EM image processing workflow for AR9 nvRNAP-Pro-cryoEM.
a, Schematic illustrating image processing jobs, software, and numbers of micrographs/particles from motion correction to the final map.
b, Representative micrograph after motion correction (our of a total of 5,139).
c, 2D class averages representing ~244,000 particles (~4,600 particles per class on average).
d, Local resolution map calculated using ResMap. The color bar gives the resolution in Å.
e, 2D histogram (heat map) of particle orientations according to their azimuth and elevation (angular distribution).
f, Fourier shell correlation (FSC) curve of the tightly masked map, with a nominal resolution of 3.80 Å.

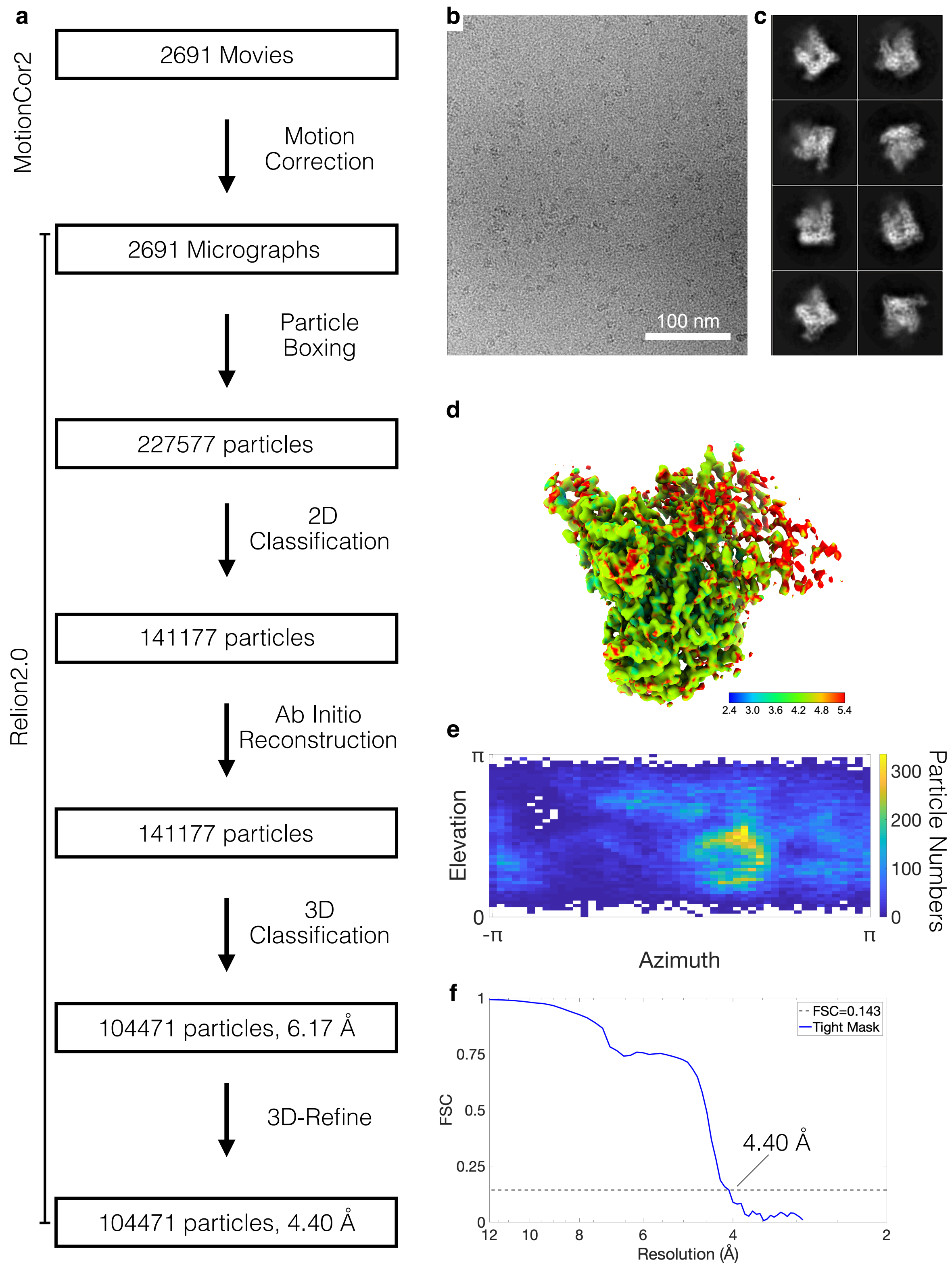

Supplementary Figure 8. Cryo-EM image processing workflow for AR9 nvRNAP-holo-cryoEM.
a, Schematic illustrating image processing jobs, software, and numbers of micrographs/particles from motion correction to the final map.
b, Representative micrograph after motion correction (out of a total of 2,633).
c, 2D class averages representing ~141,000 particles (~6,100 particles per class on average).
d, Local resolution map calculated using ResMap. The color bar gives the resolution in Å.
e, 2D histogram (heat map) of particle orientations according to their azimuth and elevation (angular distribution).
f, Fourier shell correlation (FSC) curve of the tightly masked map, with a nominal resolution of 4.40 Å.

Supplementary Table 1. Dataset names, methods, and figure and table references.

Dataset name	AR9 nvRNAP-core-Xray	AR9 nvRNAP-holo-cryoEM	AR9 nvRNAP-Pro-Xray	AR9 nvRNAP-Pro-cryoEM
Sample	AR9 nvRNAP core	AR9 nvRNAP holoenzyme	AR9 nvRNAP promoter complex	
Composition	gp105, gp089, gp270, gp154	gp105, gp089, gp270, gp154, gp226	gp105, gp089, gp270, gp154, gp226 + downstream forked nucleotide (as designed) + upstream copy of the same (fortuitous)	
Method	X-ray crystallography	Cryo-EM	X-ray crystallography	Cryo-EM
Resolution (Å)	3.30	4.40	3.38	3.80
Features	TLID is ordered in only one molecule	TLID, gp226 NTD and σ finger-like element are all disordered	The protein part is ordered almost in its entirety.<br>A large fraction of the nucleic acid is also ordered.	The protein part is ordered almost in its entirety.<br>Only three nucleotides of the promoter sequence are ordered.
Shown in Figures	4	5 (cryo-EM map only), Suppl Fig. 8d.	2a, 2c, 3a, 5 (atomic model only), 6a, 7a, 7c, 7d, 8a, 8c, 8e,<br>Suppl Figs. 2, 4a, 6.	7b, 7e, 9a,<br>Suppl Fig. 7d.
Shown in Tables	Suppl Table 2	Suppl Tables 3, 4	Suppl Table 2	Suppl Tables 3, 4
PDB and EMDB deposition codes	PDB code 7S00	PDB code 7UM1, EMDB-24765	PDB code 7S01	PDB code 7UM0, EMDB-24763

Supplementary Table 2. X-ray data collection and atomic structure refinement statistic.

X-ray dataset name and type	AR9 nvRNAP core Standard unit cell *Native*	AR9 nvRNAP core Standard unit cell *Thimerosal (Hg) derivative*	AR9 nvRNAP core Standard unit cell *Ta_6Br_{12} derivative*	AR9 nvRNAP core Large unit cell *Thimerosal (Hg) derivative*	AR9 nvRNAP Promoter complex *Native*
Reference name	AR9 nvRNAP-core-Xray				AR9 nvRNAP-Pro-Xray
Composition of asymmetric unit:					
Number of nvRNAP molecules	2	2	2	8	1
Number of protein chains	8	8	8	32	5
Number of DNA chains	0	0	0	0	4
**Data collection**					
Beamline	APS 21-ID-G	APS 21ID-F	ALS 5.0.2	APS 21-ID-D	APS 21-ID-D
Detector	Rayonix MX-300	Rayonix MX-300	Dectris Pilatus3 6M 25Hz	Dectris Eiger 9M	Dectris Eiger 9M
Wavelength (Å)	0.97857	0.97872	1.25515	1.0050	0.91840
Space group	$P2_12_12_1$	$P2_12_12_1$	$P2_12_12_1$	$P2_12_12_1$	C2
Cell dimensions					
a, *b*, *c* (Å)	112.86, 166.27, 307.22	113.43, 169.51, 308.30	112.77, 171.51, 309.76	171.24, 231.78, 592.45	176.93, 110.46, 222.38
α, β, γ (°)	90.00, 90.00, 90.00	90.00, 90.00, 90.00	90.00, 90.00, 90.00	90.00, 90.00, 90.00	90.00, 98.52, 90.00
Resolution (Å)	50.0-3.30 (3.50-3.30)*	50.0-3.60 (3.82-3.60)	50.0-4.53 (4.81-4.53)	50.0-3.79 (4.02-3.79)	50.0-3.38 (3.58-3.38)
R_{merge} (%)	12.3 (124.2)	18.2 (201.2)	20.6 (172.2)	20.9 (138.0)	15.1 (101.1)
$I / \sigma I$	10.07 (1.17)	8.77 (1.13)	6.75 (1.07)	6.22 (1.02)	7.91 (1.13)
Completeness (%)	98. 6 (98.7)	99.7 (98.5)	99.6 (98.3)	99.4 (99.7)	95.3 (85.7)
Redundancy	4.55 (4.20)	6.95 (6.87)	6.25 (6.44)	6.30 (6.18)	3.33 (3.40)
$CC_{1/2}$	99.8 (42.1)	99.8 (54.8)	99.7 (47.1)	99.5 (52.4)	99.2 (65.0)
**Refinement**					
Resolution (Å)	49.4 – 3.30				50.0 – 3.40
No. reflections	86,075				56,728
R_{work} / R_{free}	0.2180 / 0.2580				0.2379 / 0.2921
No. atoms					
Protein	33,892				21,749
Ligand/ion	2 (Zn^{2+})				1,500 (DNA) / 96 (ions)
Water	0				0
B-factors (Å^2)					
Protein	154.65				126.15
Ligand/ion	154.85				239.32 (DNA) / 157.88 (ions)
Water	NA				NA
R.m.s. deviations					
Bond lengths (Å)	0.003				0.002
Bond angles (°)	0.57				0.462
Validation					
MolProbity score	1.46				1.34
Clashscore	8.48				6.24
Poor rotamers (%)	0.03				0.00
Ramachandran plot					
Favored (%)	98.30				98.07
Allowed (%)	1.70				1.93
Disallowed (%)	0.00				0.00

*Values in parentheses are for the highest resolution shell.

Supplementary Table 3. Cryo-EM data collection and map refinement statistics.

	AR9 nvRNAP promoter complex (EMDB-24763)	AR9 nvRNAP holoenzyme (EMDB-24765)
**Data collection and processing**		
Magnification	80,000	130,000
Voltage (kV)	300	300
Electron exposure (e^{-}/Å^2)	43.7	43.2
Defocus range (μm)	−1 to −4	−1 to −3
Pixel size (Å)	1.09	1.08
Symmetry imposed	C1	C1
Initial particle images (no.)	420,791	227,577
Final particle images (no.)	106,876	104,471
Map resolution (Å)	3.8	4.4
FSC threshold	0.143	0.143
Map resolution range (Å)	2.5-5.5	3.1-6.1

Supplementary Table 4. Cryo-EM-derived atomic structure refinement statistics.

	AR9 nvRNAP promoter complex (EMDB-24763, PDB code 7UM0)		AR9 nvRNAP holoenzyme (EMDB-24765, PDB code 7UM1)	
**Model**				
Composition				
Chains	6		5	
Atoms (Hydrogens)	21760 (0)		18356 (0)	
Residues	Protein: 2647. Nucleotides: 3		Protein: 2247 Nucleotide: 0	
Water	0		0	
Ligands	Zn: 1		Zn: 1	
Bonds (RMSD)				
Length (Å) (# > 4σ)	0.002 (0)		0.005 (0)	
Angles (°) (# > 4σ)	0.448 (5)		0.861 (15)	
Molprobity score	1.54		2.23	
Clash score	7.24		22.73	
Ramachandran plot (%)				
Outliers	0.00		0.00	
Allowed	2.73		5.68	
Favored	97.27		94.32	
Ramachandran Z-score (RMSD)				
whole	-0.49 (0.16), N = 2637		-0.44 (0.17), N = 2235	
helix	0.33 (0.16), N = 1055		0.40 (0.17), N = 1001	
sheet	-0.07 (0.35), N = 251		-0.59 (0.34), N = 197	
loop	-0.86 (0.16), N = 1331		-0.85 (0.19), N = 1037	
Rotamer outliers (%)	0.12		0.82	
Cβ outliers (%)	0.00		0.00	
Peptide plane (%)				
Cis proline/general	3.0/0.0		1.7/0.0	
Twisted proline/general	0.0/0.0		0.0/0.1	
CaBLAM outliers (%)	1.83		1.66	
ADP (B-factors, $Å^2$)				
Iso/Aniso (#)	21700/60		18356/0	
min/max/mean				
Protein	29.32/242.81/88.16		37.27/225.20/120.21	
Nucleotide	86.93/86.93/86.93		---	
Ligand	76.59/108.00/85.81		193.52/193.52/193.52	
Water	---		---	
Occupancy				
Mean	1.00		1.00	
occ = 1 (%)	100.00		100.00	
0 < occ < 1 (%)	0.00		0.00	
occ > 1 (%)	0.00		0.00	
**Data**				
Box				
Lengths (Å)	144.72, 166.32, 113.40		142.56, 136.08, 115.56	
Angles (°)	90.00, 90.00, 90.00		90.00, 90.00, 90.00	
Supplied resolution (Å)	3.9		4.0	
Resolution Estimates (Å)	Masked	Unmasked	Masked	Unmasked
d FSC (half maps: 0.143)	---	---	---	---
d 99 (full/half1/half2)	4.3/---/---	4.2/---/---	4.4/---/---	4.4/---/---
d model	4.0	4.0	4.3	4.4
d FSC model (0/0.143/0.5)	3.0/3.6/4.0	3.0/3.8/4.2	4.1/4.2/4.4	4.2/4.3/4.5
Map min/max/mean	-1.29/3.51/0.13		-0.15/0.19/0.00	
**Model vs. Data**				
CC (mask)	0.85		0.78	
CC (box)	0.85		0.75	
CC (peaks)	0.77		0.65	
CC (volume)	0.83		0.77	
Mean CC for ligands	0.78		0.67	

Supplementary Table 5. Oligonucleotides used in the study.

PCR primers for site-directed mutagenesis of gp226 (Figure 8b, 8d, 8g)

Mutation	*Oligonucleotide name*	*5'-3' sequence*
Gp226 V206G	gp226-V206X-rev	aacatctgagtactttgtctcgaagacgcga
	gp226-V206G-dir	gagacaaagtactcagatgttggaatctggacgtaccttaaaaac
Gp226 A^{5} mutant (R389A, K390A, R394A, K395A, K396A)	gp226-R389X-rev	cacatttggtgcgatagcgcaggataaaatctc
	gp226-A-for	gctatcgcaccaaatgtggccgcgatgaacaacgctgcagcattagttgataaaatcatc
gp226 Y246A	gp226_Y246A_F	atctccgcgcttgacgttgtgatcaaacagaaattg
	gp226_Y246A_R	gtcaagcgcggagattaccgagctattgtgtttc
gp226 S245E	gp226_S245E_F	gtaatcgaataccttgacgttgtgatcaaacag
	gp226_S245E_R	aaggtattcgattaccgagctattgtgtttcaac

PCR primers used to create a plasmid for expression of the tagless version of AR9 nvRNAP core

	Oligonucleotide name	*5'-3' sequence*
	nvRNAP-tag-free-for-66.5	tttaactttaagaaggagatataccatggggaaaaaattatcgttaatcgatttcaac
	nvRNAP-tag-free-rev-72.1	cagcagcggtttctttaccagactcgagttatttttcatc

DNA oligonucleotides for structure determination of AR9 nvRNAP promoter complex

	Oligonucleotide name	*5'-3' sequence*
	[+3;+16] non-template strand	atcacatattggag
	[-16;+16] template strand all U	ctccaatatgtgatataatatauuguuuattg

DNA templates used to examine the dependence of *in vitro* transcription activity of the AR9 nvRNAP on the position and number of T bases in the promoter (Figure 1c)

Name of template in figure	*Oligonucleotide name*	*5'-3' sequence*
all U	[+3;+16] non-template strand	atcacatattggag
	[-16;+16] template strand all U	ctccaatatgtgatataatatauuguuuattg
(-12)T	[+3;+16] non-template strand	atcacatattggag
	[-16;+16] template strand T(-12)	ctccaatatgtgatataatatauuguutattg
(-11)T	[+3;+16] non-template strand	atcacatattggag
	[-16;+16] template strand T(-11)	ctccaatatgtgatataatatauugutuattg
(-10)T	[+3;+16] non-template strand	atcacatattggag
	[-16;+16] template strand T(-10)	ctccaatatgtgatataatatauugtuuattg
(-8)T	[+3;+16] non-template strand	atcacatattggag
	[-16;+16] template strand T(-8)	ctccaatatgtgatataatatautguuuattg
(-7)T	[+3;+16] non-template strand	atcacatattggag
	[-16;+16] template strand T(-7)	ctccaatatgtgatataatatatuguuuattg
all T	[+3;+16] non-template strand	atcacatattggag
	[-16;+16] template strand all T	ctccaatatgtgatataatatattgtttattg

DNA templates used to examine the *in vitro* transcription activity of the AR9 nvRNAP gp226 V206G mutant (Figure 8b)

Name of template in figure	*Oligonucleotide name*	*5'-3' sequence*
all U	[+3;+16] non-template strand	atcacatattggag
	[-16;+16] template strand all U	ctccaatatgtgatataatatauuguuuattg
(-11)T	[+3;+16] non-template strand	atcacatattggag
	[-16;+16] template strand T(-11)	ctccaatatgtgatataatatauugutuattg
(-10)T	[+3;+16] non-template strand	atcacatattggag
	[-16;+16] template strand T(-10)	ctccaatatgtgatataatatauugtuuattg

all T	[+3;+16] non-template strand	atcacatattggag
	[-16;+16] template strand all T	ctccaatatgtgatataatatattgtttattg

DNA templates used to examine the *in vitro* transcription activity of the AR9 nvRNAP gp226 Y246A and gp226 S245E mutants (Figure 8d)

Name of template in figure	*Oligonucleotide name*	*5'-3' sequence*
ds DNA	[-16;+16] non-template strand	caataaacaatatattatatcacatattggag
	[-16;+16] template strand all U	ctccaatatgtgatataatatauuguuuattg
fork DNA	[+3;+16] non-template strand	atcacatattggag
	[-16;+16] template strand all U	ctccaatatgtgatataatatauuguuuattg

PCR primers that were used for PCR amplification of genomic DNA fragments to examine the *in vitro* transcription activity of the A^5 mutant (Figure 8g)

Name of template in figure	*Oligonucleotide name*	*5'-3' sequence*
[-60;+80] DNA	P077-for-UP-60-63.4	taatcctcctacttatctagtctataattaattgttg
	P077-rev-ROff-80-61.9	attgcttcattaaacataaatgaagactc
[-16;+80] DNA	P077-for-UP-16-60.4	caataaacaatatattatatcacatattggagg
	P077-rev-ROff-80-61.9	attgcttcattaaacataaatgaagactc

Supplementary Table 6. Summary of MD simulation configurations and results.

Free energy term	Simulation Name	Window Number	Window Steps (×10^3)	Equilibration Steps per Window (×10^3)	Total Time (ns)	Energy Value (kcal/mol)
	DNA-RNAP Alchemical	400	40	8	64	705.7 ± 2.3
	DNA (bulk water) Alchemical	400	150	30	240	693.0 ± 0.4
It is a sum of seven energy terms with a combined (propagated) error	DNA-RNAP Constraint: r	20	600	120	48	0.3 ± 0.0
	DNA-RNAP Constraint: ϕ	20	60	12	4.8	0.3 ± 0.0
	DNA-RNAP Constraint: θ	20	300	60	24	0.9 ± 0.4
	DNA-RNAP Constraint: χ	20	60	12	4.8	0.2 ± 0.0
	DNA-RNAP Constraint: ψ	20	60	12	4.8	1.0 ± 0.0
	DNA-RNAP Constraint: ξ	20	60	12	4.8	0.9 ± 0.0
	DNA-RNAP Constraint: RMSD	20	600	120	48	3.3 ± 1.4
	DNA (bulk water) Constraint: RMSD	19	10,000	2,000	760	12.7 ± 0.1
