# Supplementary material for: Structural basis of template strand deoxyuridine promoter recognition by a viral RNA polymerase
Source: Nat Commun. 2022 Jun 20;13:3526. doi: 10.1038/s41467-022-31214-6 (PMC9209446; doi:10.1038/s41467-022-31214-6)
Supplement: Supplementary file 6 — Reporting Summary [file 41467_2022_31214_MOESM6_ESM.pdf]

nature portfolio

Corresponding author(s): Petr Leiman, Maria Sokolova, Konstantin Severinov

Last updated by author(s): 5/19/2022

Reporting Summary

Nature Portfolio wishes to improve the reproducibility of the work that we publish. This form provides structure for consistency and transparency in reporting. For further information on Nature Portfolio policies, see our Editorial Policies and the Editorial Policy Checklist.

Statistics

For all statistical analyses, confirm that the following items are present in the figure legend, table legend, main text, or Methods section.

n/a	Confirmed	
☐	☒	The exact sample size (*n*) for each experimental group/condition, given as a discrete number and unit of measurement
☐	☒	A statement on whether measurements were taken from distinct samples or whether the same sample was measured repeatedly
☒	☐	The statistical test(s) used AND whether they are one- or two-sided<br>*Only common tests should be described solely by name; describe more complex techniques in the Methods section.*
☒	☐	A description of all covariates tested
☒	☐	A description of any assumptions or corrections, such as tests of normality and adjustment for multiple comparisons
☒	☐	A full description of the statistical parameters including central tendency (e.g. means) or other basic estimates (e.g. regression coefficient) AND variation (e.g. standard deviation) or associated estimates of uncertainty (e.g. confidence intervals)
☒	☐	For null hypothesis testing, the test statistic (e.g. *F*, *t*, *r*) with confidence intervals, effect sizes, degrees of freedom and *P* value noted<br>*Give P values as exact values whenever suitable.*
☒	☐	For Bayesian analysis, information on the choice of priors and Markov chain Monte Carlo settings
☒	☐	For hierarchical and complex designs, identification of the appropriate level for tests and full reporting of outcomes
☒	☐	Estimates of effect sizes (e.g. Cohen's *d*, Pearson's *r*), indicating how they were calculated

Our web collection on statistics for biologists contains articles on many of the points above.

Software and code

Policy information about availability of computer code

Data collection	Transcription reaction results were visualized by Typhoon FLA scanner (GE Healthcare).<br>X-ray data collection: software-hardware packages that were deployed at the APS LS-CAT Sector 21 beamlines in June 2017 and at the ALS BCSB beamline 5.0.1 in February 2018.<br>Cryo-EM data collection: EPU (Themo Fisher Scientific).
Data analysis	X-ray diffraction: XDS (VERSION Jun 1, 2017 BUILT=20170615), Shelx program package (Version 2016/1), HKL2MAP (Version 0.4.b-beta), CCP4 program package (version 7.0.047), Parrot (version 1.0.4), Phaser (version 2.2,17) , Coot (versions 0.8.9 and 0.9.1), Phenix (versions 1.13-2998 and 1.20-4487), Refmac5 (version 5.8.0189), SHARP (Version 2016-01-13), Buccaneer (Version 1.5)<br>Cryo-EM: Eman2, Relion 2.0, Relion 3.0, CryoSPARC 3.0, CTFFIND4.1, E2boxer, MotionCor2, Gctf<br>Molecular dynamics: NAMD (version 2.10) , ParseFEP package, VMD<br>Macromolecular structure visualization: ChimeraX, Chimera, APBS, ResMap<br>Bioinformatics: BLAST, ESPRIT, CLUSTALX

For manuscripts utilizing custom algorithms or software that are central to the research but not yet described in published literature, software must be made available to editors and reviewers. We strongly encourage code deposition in a community repository (e.g. GitHub). See the Nature Portfolio guidelines for submitting code & software for further information.

Data

Policy information about availability of data

All manuscripts must include a data availability statement. This statement should provide the following information, where applicable:

- Accession codes, unique identifiers, or web links for publicly available datasets
- A description of any restrictions on data availability
- For clinical datasets or third party data, please ensure that the statement adheres to our policy

All macromolecular structure data generated in this study have been deposited to the Protein Data Bank and Electron Microscopy Data Bank under the following accession numbers: PDB code 7S00 [https://www.rcsb.org/structure/7S00] (AR9 nvRNAP core X-ray structure); PDB code 7S01 [https://www.rcsb.org/structure/7S01] (AR9 nvRNAP promoter complex X-ray structure); PDB code 7UM0 [https://www.rcsb.org/structure/7UM0] (AR9 nvRNAP promoter complex cryo-EM structure); PDB code 7UM1 [https://www.rcsb.org/structure/7UM1] (AR9 nvRNAP holoenzyme cryo-EM structure); EMDB code EMD-24763 [https://www.ebi.ac.uk/emdb/EMD-24763] (AR9 nvRNAP promoter complex cryo-EM density); EMDB code EMD-24765 [https://www.ebi.ac.uk/emdb/EMD-24765] (AR9 nvRNAP holoenzyme cryo-EM density).

The unedited images of the in vitro transcription assays and polyacrylamide gel photographs can be found in the Source Data File 1. The coordinates of the phiKZ gp68 model created by AlphaFold Colab are in the Source Data File 2. The molecular dynamics setup files and coordinates are in the Source Data File 3.

Publicly available protein atomic models with the following PDB codes were used in the study: 4AYB [https://www.rcsb.org/structure/4AYB] (Archaeal RNA Polymerase), 5ZX3 [https://www.rcsb.org/structure/5ZX3] (Mycobacterium tuberculosis RNA polymerase holoenzyme with ECF sigma factor sigma H), 6C9Y [https://www.rcsb.org/structure/6C9Y] (Cryo-EM structure of E. coli RNAP sigma70 holoenzyme), 5IPM [https://www.rcsb.org/structure/5IPM] (SigmaS-transcription initiation complex with 4-nt nascent RNA), 6JBQ [https://www.rcsb.org/structure/6JBQ] (CryoEM structure of Escherichia coli sigmaE transcription initiation complex containing 5nt of RNA), and 7OGP [https://www.rcsb.org/structure/7OGP] (Structure of the apo-state of the bacteriophage PhiKZ non-virion RNA polymerase).

Field-specific reporting

Please select the one below that is the best fit for your research. If you are not sure, read the appropriate sections before making your selection.

- [x] Life sciences
- [ ] Behavioural & social sciences
- [ ] Ecological, evolutionary & environmental sciences

For a reference copy of the document with all sections, see nature.com/documents/nr-reporting-summary-flat.pdf

Life sciences study design

All studies must disclose on these points even when the disclosure is negative.

Sample size	For macromolecular structural studies, the sample size was chosen so that reliable multiple measurements for each parameter were obtained: at least three measurements (on average) for each diffraction intensity in crystallography or ten views (on average) for each orientation in cryo-electron microscopy. Each dataset contained hundreds of thousands data points that were recorded for billions of molecules contained in each sample.<br>For in vitro transcription assays and protein domain analysis by partial proteolysis, the sample size (i. e. the volume of the reaction mixture) was chosen so that the reaction products could be visualized by autoradiography and polyacrylamide gel electrophoresis by standard tools and techniques.
Data exclusions	None
Replication	Biological specimens were prepared de novo at least twice for every experiment. Each experiment was repeated at least twice. Similar outcomes were obtained in all cases. A representative was chosen to be shown in the manuscript.
Randomization	No randomization was employed as each sample contained billions of objects (macromolecules).
Blinding	The investigators were not blinded to allocation during experiments because the subjects of the experiments (biological molecules) have no free will and cannot influence the outcome of the experiments.

Reporting for specific materials, systems and methods

We require information from authors about some types of materials, experimental systems and methods used in many studies. Here, indicate whether each material, system or method listed is relevant to your study. If you are not sure if a list item applies to your research, read the appropriate section before selecting a response.

Materials & experimental systems

n/a	Involved in the study
☒	☐ Antibodies
☒	☐ Eukaryotic cell lines
☒	☐ Palaeontology and archaeology
☒	☐ Animals and other organisms
☒	☐ Human research participants
☒	☐ Clinical data
☒	☐ Dual use research of concern

Methods

n/a	Involved in the study
☒	☐ ChIP-seq
☒	☐ Flow cytometry
☒	☐ MRI-based neuroimaging
